# Comparing clinical and genomic features based on the tumor location in patients with resected pancreatic cancer

**DOI:** 10.1186/s12885-024-12795-5

**Published:** 2024-08-26

**Authors:** Won-Gun Yun, Daeun Kim, Mirang Lee, Youngmin Han, Yoon Soo Chae, Hye-Sol Jung, Young Jae Cho, Wooil Kwon, Joon Seong Park, Daechan Park, Jin-Young Jang

**Affiliations:** 1 Department of Surgery and Cancer Research Institute, Seoul National University College of Medicine, 101 Daehak-ro, Jongno-gu, Seoul, 03080 Republic of Korea; 2 Department of Molecular Science and Technology, Ajou University, Suwon, 16499 Republic of Korea; 3 Ajou Energy Science Research Center, Ajou University, Suwon, 16499 Republic of Korea; 4 Advanced College of Bio-Convergence Engineering, Ajou University, Suwon, 16499 Republic of Korea

**Keywords:** Pancreatic neoplasms, Tumor location, Prognosis, DNA mutation analysis, Molecular subtype, Tumor microenvironment

## Abstract

**Background:**

Pancreatic cancer is anatomically divided into pancreatic head and body/tail cancers, and some studies have reported differences in prognosis. However, whether this discrepancy is induced from the difference of tumor biology is hotly debated. Therefore, we aimed to evaluate the differences in clinical outcomes and tumor biology depending on the tumor location.

**Methods:**

In this retrospective cohort study, we identified 800 patients with pancreatic ductal adenocarcinoma who had undergone upfront curative-intent surgery. Cox regression analysis was performed to explore the prognostic impact of the tumor location. Among them, 153 patients with sufficient tumor tissue and blood samples who provided informed consent for next-generation sequencing were selected as the cohort for genomic analysis.

**Results:**

Out of the 800 patients, 500 (62.5%) had pancreatic head cancer, and 300 (37.5%) had body/tail cancer. Tumor location in the body/tail of the pancreas was not identified as a significant predictor of survival outcomes compared to that in the head in multivariate analysis (hazard ratio, 0.94; 95% confidence interval, 0.77–1.14; *P* = 0.511). Additionally, in the genomic analyses of 153 patients, there were no significant differences in mutational landscapes, distribution of subtypes based on transcriptomic profiling, and estimated infiltration levels of various immune cells between pancreatic head and body/tail cancers.

**Conclusions:**

We could not find differences in prognosis and tumor biology depending on tumor location in pancreatic ductal adenocarcinoma. Discrepancies in prognosis may represent a combination of lead time, selection bias, and clinical differences, including the surgical burden between tumor sites.

**Supplementary Information:**

The online version contains supplementary material available at 10.1186/s12885-024-12795-5.

## Introduction

Pancreatic ductal adenocarcinoma (PDAC) is a leading cause of cancer-related deaths worldwide and has been characterized by dismal prognosis [1]. Regarding tumor location, PDAC was generally divided into pancreatic head and body/tail cancer. Therefore, the type of surgery that remains the backbone of treatment varies depending on the tumor location. Tumors located to the right of the superior mesenteric vein are considered pancreatic head cancers that are potentially suitable for pancreaticoduodenectomy, and tumors located to the left of the superior mesenteric vein are considered body/tail cancers that are potentially suitable for distal pancreatectomy [2].

In addition to differences in surgical methods, part of the pancreatic head, including the uncinate process, has a different embryonic origin. Because of this embryological difference, the head and body/tail of the pancreas have different innervations, blood supplies, and lymphatic and venous drainage. From a clinical perspective, pancreatic head cancer, which can induce biliary obstruction, is typically diagnosed at an early stage compared to body/tail cancer [3]. From these backgrounds, there has been a long debate regarding whether there is a real difference in prognosis between pancreatic head and body/tail cancers. Artinyan et al. (2008), using Surveillance, Epidemiology, and End Results registry, reported that pancreatic body/tail cancer had a lower resection rate and worse outcomes than those of head cancer [4]. Winer et al. (2019), using the National Cancer Database, reported that cancer localized to the pancreatic head had worse outcomes than those of body/tail cancers [5]. These contradictory findings call for further investigations because they raise the possibility that either lead time and selection bias played a role or that biological differences exist.

The recent large-scale sequencing studies have demonstrated the inter-tumoral and intra-tumoral genomic heterogeneity of PDAC [6]. With frequent alterations in main driver genes, including *KRAS*, *TP53*, *CDKN2A*, and *SMAD4*, Moffitt et al. (2015) and Bailey et al. (2016) established the molecular subtypes of PDAC, which showed the difference in survival according to tumor biology [7–9]. Some studies have noted genetic differences between pancreatic head and body/tail cancer, but most of them have flaws in that clinical information is either not taken sufficiently into account or data from various institutions are combined, making it challenging to completely rule out the impact of batch effects as well as different clinical stage and management [10–12]. Therefore, we aimed to evaluate the impact of tumor location on prognosis with consideration of other clinical and pathologic confounding factors. In addition, we aimed to explore the differences in tumor biology depending on tumor location using uniformly generated sequencing data with clinical details.

## Materials and methods

### Patient cohort

**Fig. 1:**
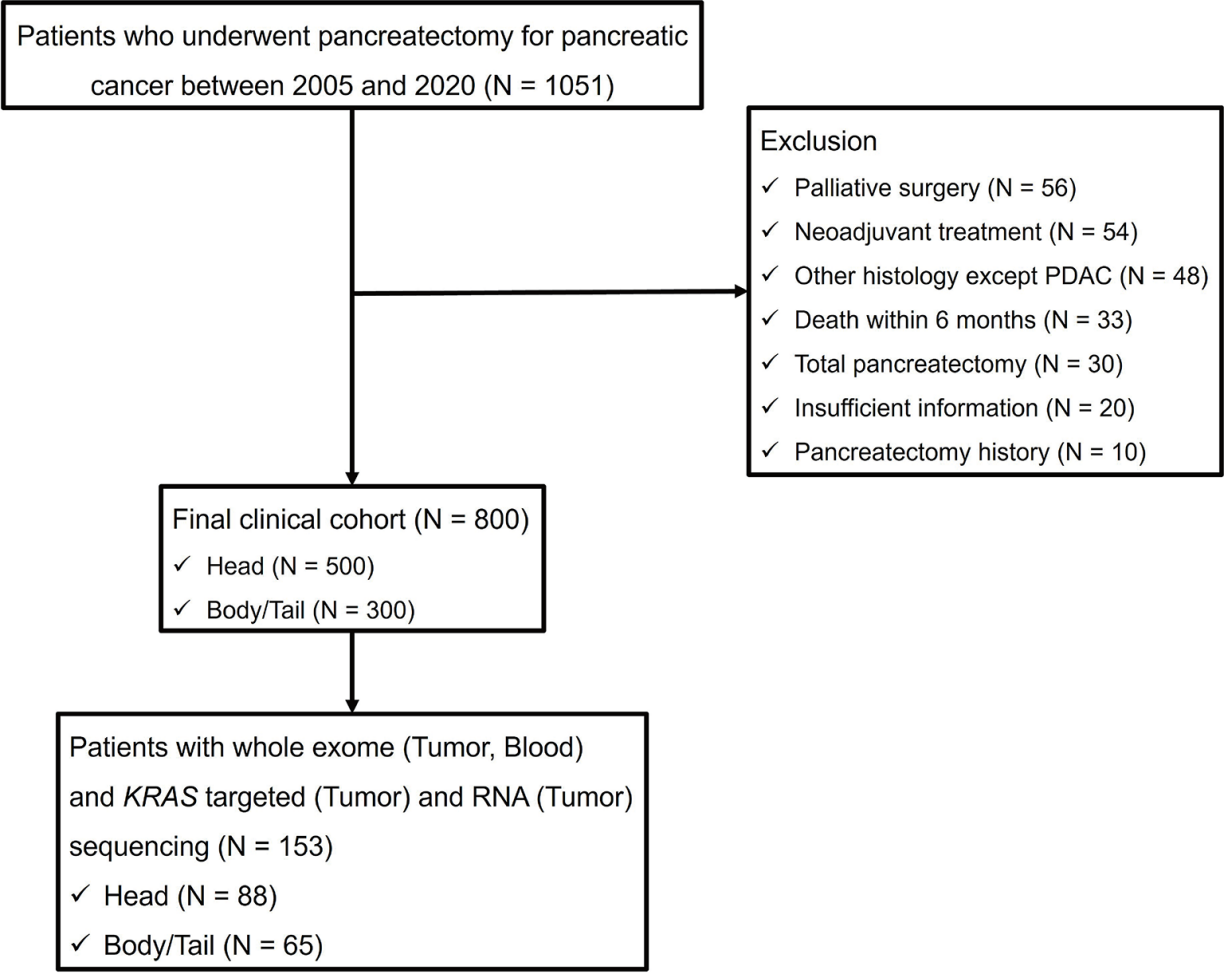
Flow chart showing pancreatic cancer patient cohorts

This retrospective cohort study was approved by the Institutional Review Board of Seoul National University Hospital (H-2309-159-1471). This study was conducted in accordance with the 1975 Declaration of Helsinki and its later versions. This work was registered at Research Registry (researchregistry9782). Data from patients who underwent pancreatectomy for pancreatic cancer between January 2005 and December 2020 were retrieved from prospectively maintained databases. Patients who underwent palliative surgery, received neoadjuvant treatment, had histology other than adenocarcinoma, underwent total pancreatectomy, lacked sufficient clinical information, had a previous history of pancreatectomy, or died within six months after surgery were excluded. Subsequently, 800 qualifying patients were selected as the cohort for analysis of clinical aspects (the entire cohort). Among them, 153 patients with sufficient tumor tissue and blood samples and who provided informed consent for next generation sequencing were selected as the cohort for the analysis of genomic aspects (genomic cohort). The genomic analysis cohort was designed to avoid discrepancies in clinical characteristics between patients with pancreatic head cancer and those with pancreatic body/tail cancer. A flow diagram of this study is presented in Fig. 1.

### Clinical data collection

Detailed clinical characteristics, including demographics, pathology, and treatment information, were collected. Patient demographics included age, sex, American Society of Anesthesiologists physical status classification, presence of symptom, and carbohydrate antigen 19 − 9 at the time before surgery. Because the period included in this study was so extensive, we classified the years 2005 to 2012 as Phase I and the years 2013 to 2020 as Phase II, depending on the time of surgery, to adjust the chronological bias. A clear resection margin was defined as the presence of tumor cells 1 mm away from any margin or circumferential surface. The treatment information included details regarding the operation and adjuvant treatment. In particular, data on both the initiation and completion of adjuvant chemotherapy were collected to evaluate the actual impact of adjuvant chemotherapy and the surgical burden. Adjuvant chemotherapy for a period of 6 months was usually recommended to all patients with appropriate physical status. Completion of adjuvant chemotherapy was defined the completion of scheduled adjuvant chemotherapy regardless of the delay. Medical oncologists made the decision to reduce the chemotherapy dose based on adverse effects, and dose reduction was not one of the requirements for completion of adjuvant chemotherapy. Patients with a microscopically positive resection margin or metastatic lymph nodes who had a high chance of recurrence were the primary candidates for adjuvant radiotherapy. Usual prescription of adjuvant radiotherapy was 50.0 Gy to the tumor bed and 45.0 Gy to the regional lymphatics in 25 fractions using simultaneous integrated boost for the concurrent chemo-radiotherapy. Follow-up data were also retrieved to evaluate the prognosis. Overall survival (OS) was measured from the date of pancreatectomy until death or the last hospital visit.

### Sample preparation for next generation sequencing

Tumor and blood samples were collected from patients who were admitted for surgery and provided informed consent for next-generation sequencing. Blood samples were collected one day before surgery, and tumor samples were collected immediately after surgical resection. Subsequently, the samples were placed in RNA later within 15 min to preserve RNA integrity and stored at -80 °C. DNA and RNA were extracted according to the Allspin (GeneAll) total DNA/RNA purification protocol.

### Whole exome sequencing

Libraries were prepared using the SureSelect Human All Exon v5 probe set, based on the Agilent SureSelect Target Enrichment protocol (version B. June 3, 2015). The libraries were loaded onto an Illumina HiSeq 2500 platform for 101-bp paired-end sequencing. Sequencing depths were set to a minimum of 100X and 300X for the blood and tumor tissue samples, respectively. The sequencing reads were mapped to the human reference genome (GRCh38) through the Burrows–Wheeler Aligner [13]. The mapping result files were further pre-processed using the Genome Analysis Tool Kit [14]. Somatic variant calling with paired tumor tissues and blood samples per patient was performed using Mutect2 according to the Genome Analysis Tool Kit best practice [15]. The somatic variants were annotated using ANNOVAR, and annotated tables were converted to mutation annotation format [16]. The R package maftools was used to analyze and visualize the mutational information.

### *KRAS* targeted sequencing

Due to the notably lower *KRAS* mutation frequency (73.1%) observed in whole exome sequencing in comparison to the previously reported rate (90% or higher), *KRAS* amplicons were sequenced at over 1,000,000X depth targeting the previously reported mutational hotspots, aiming to counteract the impact of low tumor purity [9, 17, 18]. All libraries were sequenced using the Illumina NovaSeq platform to generate paired-end 151 base pairs reads.

### RNA sequencing

RNA exome capture sequencing of tumor tissue samples was performed instead of Total RNA sequencing to overcome RNA degradation caused by pancreatic enzymes. Total RNA was quantified using Quant-IT RiboGreen. Subsequently, the mRNA-encoding exome was extracted from > 100 ng of total RNA using an Illumina TruSeq RNA exome. The cDNA library was constructed by adaptor ligation and loaded onto an Illumina HiSeq 2500 platform for 101-bp paired-end sequencing. The sequencing reads were pre-processed with Trimmomatic to remove reads containing low-quality bases and adapters [19]. Trimmed reads were mapped to GRCh38 using STAR [20]. The mapped result files were processed to quantify the expression levels of genes according to GENCODE v27 GTF annotation using RSEM [21].

### Transcriptomic profiling

To compare the distribution of previously known transcriptome-based subtypes with clinical significance between pancreatic head and body/tail cancers, the R package ConsensusClusterPlus was employed [22]. The classification of samples was based on molecular signatures defined in Bailey et al. (2016) and Moffitt et al. (2015) [8, 9].

### Deconvolution analysis

To compare the characteristics of the tumor microenvironment between pancreatic head and body/tail cancer, deconvolution analysis was performed using the Tumor Immune Estimation Resource version 2.0, a web-based tool [23]. The analysis estimated immune cell infiltration levels, including B cells, CD4 + T cells, CD8 + T cells, neutrophils, macrophages, and myeloid dendritic cells, with transcripts per million values.

### Statistical analysis

Categorical variables were expressed as numbers with percentages, while continuous variables were expressed as median values with interquartile ranges. To compare clinical characteristics according to tumor location, the chi-square and Fisher’s exact tests were used for categorical variables, and the independent t-test was used for continuous variables. Cox proportional hazards regression models, for calculating hazard ratios (HR) and 95% confidence intervals (CI), were employed to explore prognostic factors. Variables previously identified as prognostic factors were selected for multivariate analysis. Survival analysis was performed using Kaplan–Meier estimates and compared using the log–rank test. Statistical significance was set at *P* < 0.05. All statistical analyses were performed using the R software, version 4.2.3 (R Foundation for Statistical Computing).

## Results

### Clinical characteristics of entire cohort

The baseline characteristics of the entire cohort, stratified by tumor location, are summarized in Table 1. Out of the 800 patients, 500 (62.5%) had pancreatic head cancer and 300 (37.5%) had pancreatic body/tail cancer. Patients with pancreatic body/tail cancer were older than those with pancreatic head cancer (*P* < 0.001). There were no significant differences in sex and preoperative physical status between the two groups.

**Table 1:** Clinical characteristics of the entire cohort

Variables	Head	Body/Tail	*P*
Number	500	300	
Age (years), median (IQR)	65.0 (58.0–72.0)	67.0 (60.0–74.0)	< 0.001
Sex (Male)	293 (58.6)	175 (58.3)	> 0.99
ASA classification			0.949
I / II	457 (91.4)	273 (91.0)	
III / IV	43 (8.6)	27 (9.0)	
Symptom (Y)	397 (79.4)	131 (43.7)	< 0.001
CA 19 − 9 > 150 U/mL	256 (51.2)	123 (41.0)	0.006
Period			0.033
Phase I	192 (38.4)	92 (30.7)	
Phase II	308 (61.6)	208 (69.3)	
Operation			NA
PD	500 (100.0)	0 (0.0)	
DP/STP (conventional)	0 (0.0)	252 (84.0)	
DP/STP (RAMPS)	0 (0.0)	46 (15.3)	
CP	0 (0.0)	2 (0.7)	
Operation type			< 0.001
Open	471 (94.2)	233 (77.7)	
Minimally invasive	29 (5.8)	67 (22.3)	
Operation time (minute)	325 (260–385)	160 (125–191)	< 0.001
Estimated blood loss (mL)	400 (250–550)	200 (100–400)	< 0.001
Complication (Y)	73 (14.6)	23 (7.7)	0.005
Examined lymph nodes > 15	300 (60.0%)	119 (39.7%)	< 0.001
Stage			0.472
I	168 (33.6)	104 (34.3)	
II	235 (47.0)	150 (49.7)	
III	97 (19.4)	48 (16.0)	
R status (R0)	338 (67.6)	175 (58.3)	0.010
Lymphatic invasion (Y)	277 (55.4)	107 (35.7)	< 0.001
Venous invasion (Y)	214 (42.8)	135 (45.0)	0.594
Perineural invasion (Y)	446 (89.2)	239 (79.7)	< 0.001
Initiation of adjuvant CTx. (Y)	397 (79.4)	242 (80.7)	0.733
Completion of adjuvant CTx. (Y)	256 (51.2)	180 (60.0)	0.019
Adjuvant RTx. (Y)	295 (59.0)	160 (53.3)	0.135

IQR, interquartile range; ASA, American society of anesthesiologists; CA 19 − 9, carbohydrate antigen 19 − 9; PD, pancreaticoduodenectomy; DP, distal pancreatectomy; STP, subtotal pancreatectomy; RAMPS, radical antegrade modular pancreatosplenectomy; CP, central pancreatectomy; NA, not applicable; CTx, chemotherapy; RTx, radiotherapy

Regarding operation, there were several disparities between the pancreatic head and body/tail cancer groups. All patients with pancreatic head cancer underwent pancreaticoduodenectomy, whereas most patients with pancreatic body/tail cancer (298/300, 99.3%) underwent distal or subtotal pancreatectomy without reconstruction. Minimally invasive surgery was performed more frequently for pancreatic body/tail (22.3%) cancers than for pancreatic head (5.8%) cancers (*P* < 0.001). Compared to patients with pancreatic head cancer, those with pancreatic body/tail cancer experienced a much shorter operation time and lower intraoperative blood loss.

There were no discernible differences in the rates of initiating adjuvant chemotherapy between patients with pancreatic head (79.4%) and body/tail (80.7%) cancers (*P* = 0.733). However, the completion rate of adjuvant chemotherapy was noticeably higher in patients with pancreatic body/tail cancer (60.0%) than in those with pancreatic head cancer (51.2%; *P* = 0.019).

To evaluate the differences in baseline characteristics according to the treatment period, the baseline characteristics stratified by treatment period are summarized in Supplementary Table 1. There were no differences in the initiation and completion rates of adjuvant chemotherapy between phase I and phase II, however there were more patients with body/tail cancer and those who underwent minimally invasive surgery in phase II compared to phase I.

### Prognostic power of tumor location

Survival and univariate and multivariate Cox regression analyses were performed for the entire cohort (Table 2). In survival and univariable analysis, almost all variables including tumor location (Median OS, 27 vs. 39 months; HR, 0.74; 95% CI, 0.62–0.88; *P* < 0.001; head vs. body/tail), non-initiation (Median OS, 35 vs. 16 months; HR, 2.10; 95% CI, 1.74–2.54; *P* < 0.001) and non-completion (Median OS, 53 vs. 17 months; HR, 3.08; 95% CI, 2.61–3.64; *P* < 0.001) of adjuvant chemotherapy were identified as significant prognostic factors. However, multivariable analysis adjusting for confounding variables revealed that the tumor location was not the independent prognostic factor (HR, 0.94; 95% CI, 0.77–1.14; *P* = 0.511; body/tail compared with head). Regarding adjuvant chemotherapy, non-initiation (HR, 0.92; 95% CI, 0.70–1.21; *P* = 0.556) of treatment was not identified as a prognostic factor, whereas non-completion (HR, 3.16; 95% CI, 2.59–3.86; *P* < 0.001) of treatment was identified as the most powerful prognostic factor among several variables.

**Table 2:** Prognostic factors for overall survival in the entire cohort, based on Cox-regression models

Variables	Median OS (95% CI)	Univariate analysis		Multivariate analysis	
		HR (95% CI)	*P*	HR (95% CI)	*P*
Age (years)					
> 65	28 (25, 31)	1.00 (Ref)		1.00 (Ref)	
≤ 65	33 (30, 39)	0.82 (0.70, 0.97)	0.021	0.81 (0.68, 0.97)	0.021
Sex					
Male	28 (26, 31)	1.00 (Ref)		1.00 (Ref)	
Female	34 (30, 40)	0.81 (0.69, 0.96)	0.014	0.76 (0.64, 0.90)	0.002
ASA classification					
I / II	30 (28, 34)	1.00 (Ref)		1.00 (Ref)	
III / IV	29 (25, 40)	1.21 (0.92, 1.59)	0.178	0.91 (0.68, 1.21)	0.507
Symptom					
Y	27 (25, 31)	1.00 (Ref)		1.00 (Ref)	
N	37 (30, 48)	0.73 (0.61, 0.87)	< 0.001	0.89 (0.73, 1.09)	0.272
CA 19 − 9 (U/mL)					
> 150	25 (23, 28)	1.00 (Ref)		1.00 (Ref)	
≤ 150	39 (33, 47)	0.61 (0.52, 0.72)	< 0.001	0.66 (0.56, 0.78)	< 0.001
Period					
Phase I	25 (21, 30)	1.00 (Ref)		1.00 (Ref)	
Phase II	35 (30, 42)	0.67 (0.57, 0.79)	< 0.001	0.59 (0.48, 0.71)	< 0.001
Location					
Head	27 (25, 30)	1.00 (Ref)		1.00 (Ref)	
Body/Tail	39 (32, 51)	0.74 (0.62, 0.88)	< 0.001	0.96 (0.78, 1.17)	0.675
Operation type					
Open	26 (29, 31)	1.00 (Ref)		1.00 (Ref)	
MIS	55 (45, NR)	0.54 (0.40, 0.73)	< 0.001	0.77 (0.56, 1.07)	0.115
Complication					
Y	31 (29, 34)	1.00 (Ref)		1.00 (Ref)	
N	24 (20, 36)	1.13 (0.88, 1.45)	0.340	1.13 (0.87, 1.45)	0.362
ELN					
> 15	29 (26, 32)	1.00 (Ref)		1.00 (Ref)	
≤ 15	32 (29, 37)	0.84 (0.72, 0.99)	0.041	0.94 (0.79, 1.12)	0.466
Stage					
I	54 (44, 67)	1.00 (Ref)		1.00 (Ref)	
II	28 (25, 31)	1.67 (1.38, 2.02)	< 0.001	1.27 (1.03, 1.57)	0.029
III	18 (16, 24)	2.70 (2.15, 3.41)	< 0.001	1.87 (1.43, 2.45)	< 0.001
R status					
R0	32 (29, 37)	1.00 (Ref)		1.00 (Ref)	
R1	26 (23, 31)	1.31 (1.10, 1.55)	0.002	1.15 (0.96, 1.39)	0.132
Lymphatic inv.					
Y	24 (21, 27)	1.00 (Ref)		1.00 (Ref)	
N	40 (35, 48)	0.58 (0.49, 0.69)	< 0.001	0.65 (0.54, 0.78)	< 0.001
Venous inv.					
Y	25 (23, 27)	1.00 (Ref)		1.00 (Ref)	
N	39 (34, 45)	0.61 (0.51, 0.71)	< 0.001	0.77 (0.64, 0.93)	0.006
Perineural inv.					
Y	28 (26, 31)	1.00 (Ref)		1.00 (Ref)	
N	55 (38, 81)	0.54 (0.42, 0.70)	< 0.001	0.78 (0.60, 1.03)	0.082
Initiation of AC					
Y	35 (32, 40)	1.00 (Ref)		1.00 (Ref)	
N	16 (13, 22)	2.10 (1.74, 2.54)	< 0.001	0.91 (0.69, 1.20)	0.503
Completion of AC					
Y	53 (46, 60)	1.00 (Ref)		1.00 (Ref)	
N	17 (15, 19)	3.08 (2.61, 3.64)	< 0.001	3.19 (2.61, 3.90)	< 0.001
Adjuvant RTx.					
Y	36 (33, 42)	1.00 (Ref)		1.00 (Ref)	
N	25 (23, 28)	1.41 (1.19, 1.66)	< 0.001	1.17 (0.94, 1.46)	0.163

OS, overall survival; HR, hazard ratio; CI, confidence interval; Ref, reference; ASA, American society of anesthesiologists; CA 19 − 9, carbohydrate antigen 19 − 9; MIS, minimally invasive surgery; ELN, examined lymph nodes; inv, invasion; AC, adjuvant chemotherapy; CTx, chemotherapy; RTx, radiotherapy

### Clinical characteristics of genomic cohort

The baseline characteristics of the genomic cohort stratified by tumor location are summarized in Table 3. Out of the 153 patients, 88 (57.5%) had pancreatic head cancer and 65 (42.5%) had pancreatic body/tail cancer. There were no significant differences in demographics, including age, sex, and preoperative physical status between the two groups. In addition, the completion rates of adjuvant chemotherapy, which was the most powerful prognostic factor in the entire cohort, were almost the same between patients with pancreatic head cancer (46.6%) and those with pancreatic body/tail cancer (47.7%; *P* > 0.99). Even when compared to the entire cohort, there were no discernible differences in the frequencies of prognostic variables including pathological stage and adjuvant chemotherapy within the genomic cohort.

**Table 3:** Clinical characteristics of the genomic cohort

Variables	Head	Body/Tail	*P*
Number	88	65	
Age (years), median (IQR)	66.5 (57.8–72.0)	67.0 (60.0–74.0)	0.421
Sex (Male)	51 (58.0)	34 (52.3)	0.596
ASA classification			> 0.99
I / II	82 (93.2)	61 (93.8)	
III / IV	6 (6.8)	4 (6.2)	
Symptom (Y)	70 (79.5)	30 (46.2)	< 0.001
CA 19 − 9 > 150 U/mL	42 (47.7)	35 (53.8)	0.559
Period			> 0.99
Phase I	30 (34.1)	23 (35.4)	
Phase II	58 (65.9)	42 (64.6)	
Operation			NA
PD	88 (100.0)	0 (0.0)	
DP/STP (conventional)	0 (0.0)	57 (87.7)	
DP/STP (RAMPS)	0 (0.0)	8 (12.3)	
Operation type			0.002
Open	55 (100.0)	58 (89.2)	
Minimally invasive	0 (0.0)	7 (10.8)	
Operation time (minute)	322 (269–376)	170 (140–215)	< 0.001
Estimated blood loss (mL)	375 (250–500)	200 (100–400)	0.030
Complication (Y)	6 (6.8)	5 (7.7)	> 0.99
Examined lymph nodes > 15	50 (56.8%)	27 (41.5%)	0.088
Stage			0.676
I	21 (23.9)	17 (26.2)	
II	47 (53.4)	37 (56.9)	
III	20 (22.7)	11 (16.9)	
R status (R0)	63 (71.6)	38 (58.5)	0.128
Lymphatic invasion (Y)	52 (59.1)	22 (33.8)	0.003
Venous invasion (Y)	45 (51.1)	31 (47.7)	0.797
Perineural invasion (Y)	83 (94.3)	49 (75.4)	0.002
Initiation of adjuvant CTx. (Y)	68 (77.3)	50 (76.9)	> 0.99
Completion of adjuvant CTx. (Y)	41 (46.6)	31 (47.7)	> 0.99
Adjuvant RTx. (Y)	48 (54.5)	33 (50.8)	0.765

IQR, interquartile range; ASA, American society of anesthesiologists; CA 19 − 9, carbohydrate antigen 19 − 9; PD, pancreaticoduodenectomy; DP, distal pancreatectomy; STP, subtotal pancreatectomy; CTx, chemotherapy; RTx, radiotherapy

### Mutational landscape

**Fig. 2:**
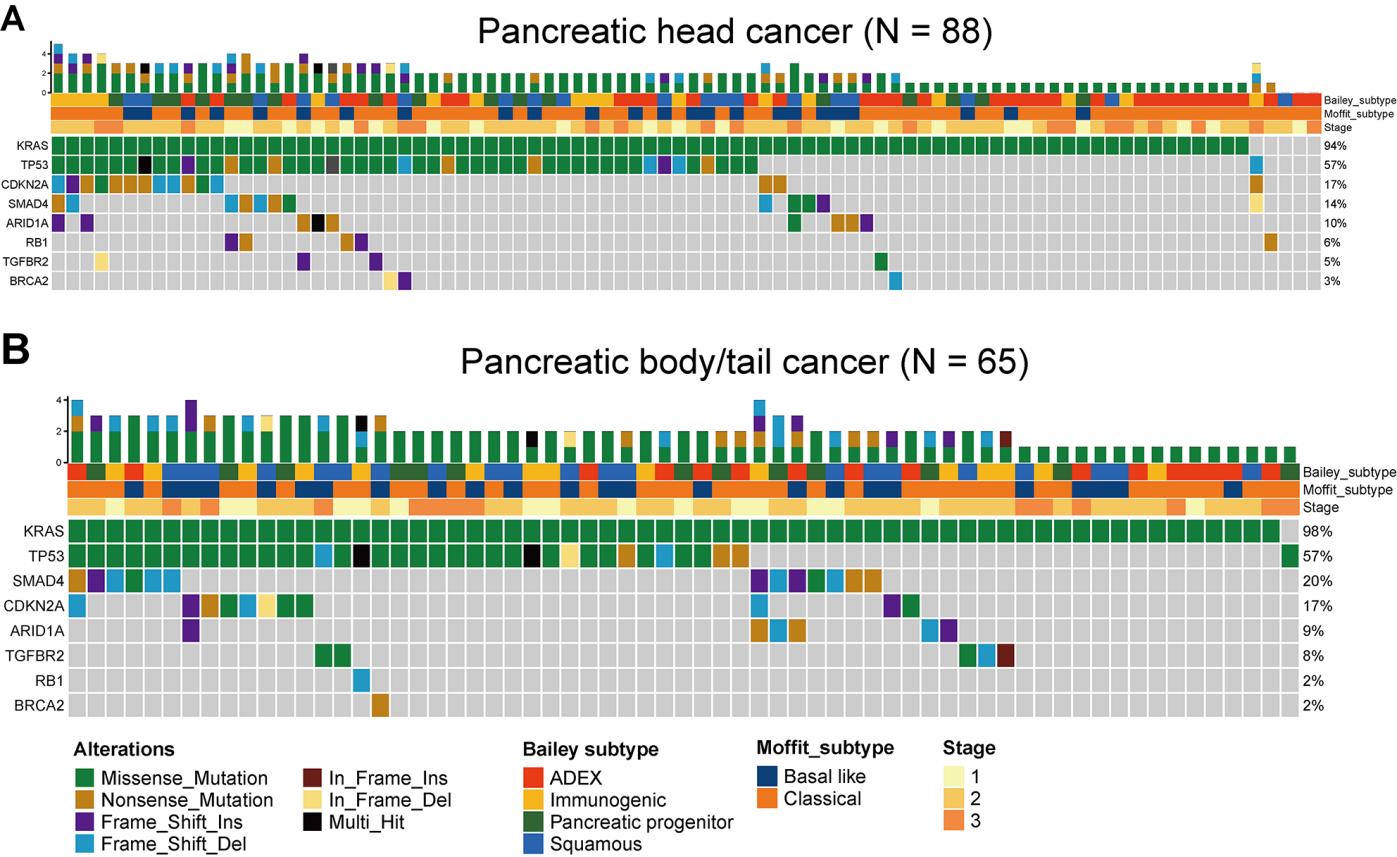
Mutational landscape in patients with pancreatic (**A**) head and (**B**) body/tail cancer

The mutational status of the genomic cohort is shown in Fig. 2. The four most common genomic alterations were *KRAS* (targeted sequencing), *TP53*, *CDKN2A*, and *SMAD4*, which are known to be the main driver gene alterations, in patients with both pancreatic head and body/tail cancers. There were no significant differences in the frequency of common genomic alterations between the two groups (Table 4).

**Table 4:** Comparison of frequency of common genomic alterations according to the tumor location

Variables	Head	Body/Tail	*P*
Number	88	65	
*KRAS*			0.242
Wild type	5 (5.7)	1 (1.5)	
Mutant	83 (94.3)	64 (98.5)	
*TP53*			> 0.99
Wild type	37 (42.5)	28 (43.1)	
Mutant	50 (57.5)	37 (56.9)	
*CDKN2A*			> 0.99
Wild type	73 (83.0)	54 (83.1)	
Mutant	15 (17.0)	11 (16.9)	
*SMAD4*			0.406
Wild type	76 (86.4)	52 (80.0)	
Mutant	12 (13.6)	13 (20.0)	
*ARID1A*			> 0.99
Wild type	79 (89.8)	59 (90.8)	
Mutant	9 (10.2)	6 (9.2)	
*TGFBR2*			0.496
Wild type	84 (95.5)	60 (92.3)	
Mutant	4 (4.5)	5 (7.7)	
*RB1*			0.242
Wild type	5 (5.7)	64 (98.5)	
Mutant	83 (94.3)	1 (1.5)	
*BRCA2*			0.637
Wild type	85 (96.6)	64 (98.5)	
Mutant	3 (3.4)	1 (1.5)	

### Comparison based on gene expression

**Fig. 3:**
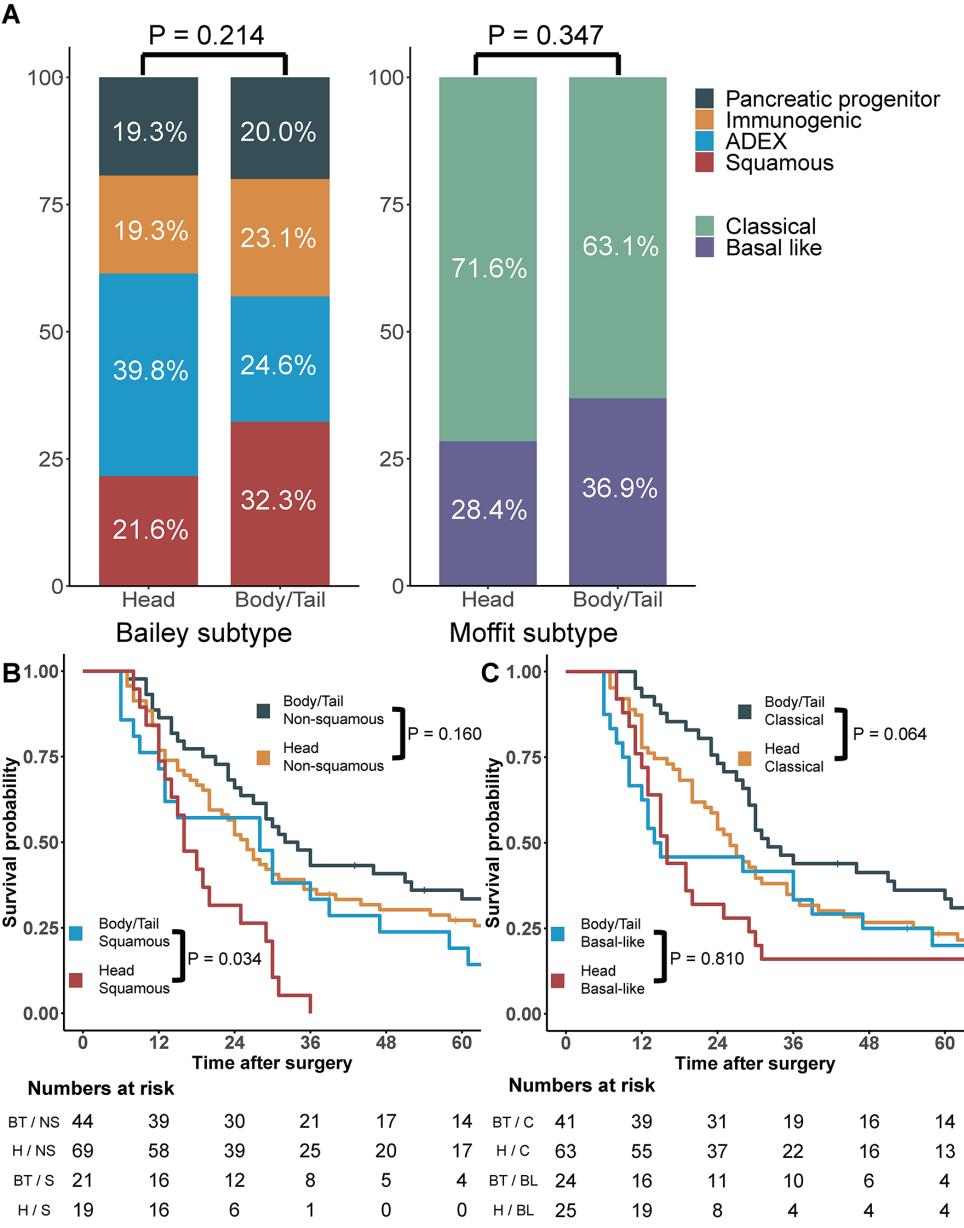
Subtypes based on transcriptomic profiling. (**A**) Distribution of subtypes according to tumor location. (**B**) Overall survival according to subtypes defined by Bailey et al. (2016) and tumor location. (**C**) Overall survival according to subtypes defined by Moffitt et al. (2015) and tumor location

While molecular subtypes associated with poor prognosis, such as the squamous subtype described by Bailey et al. (2016) and basal-like subtype described by Moffitt et al. (2015), demonstrated higher ratios in patients with pancreatic body/tail cancer than in those with pancreatic head cancer, these findings were not statistically significant (Fig. 3A) [8, 9]. Considering these molecular subtypes, survival analyses according to tumor location were performed (Fig. 3B–C). There were no significant differences in OS between patients with pancreatic head cancer and those with pancreatic body/tail cancer in almost all subgroups stratified by molecular subtype, except the squamous subtype.

### Immune cell infiltration

**Fig. 4:**
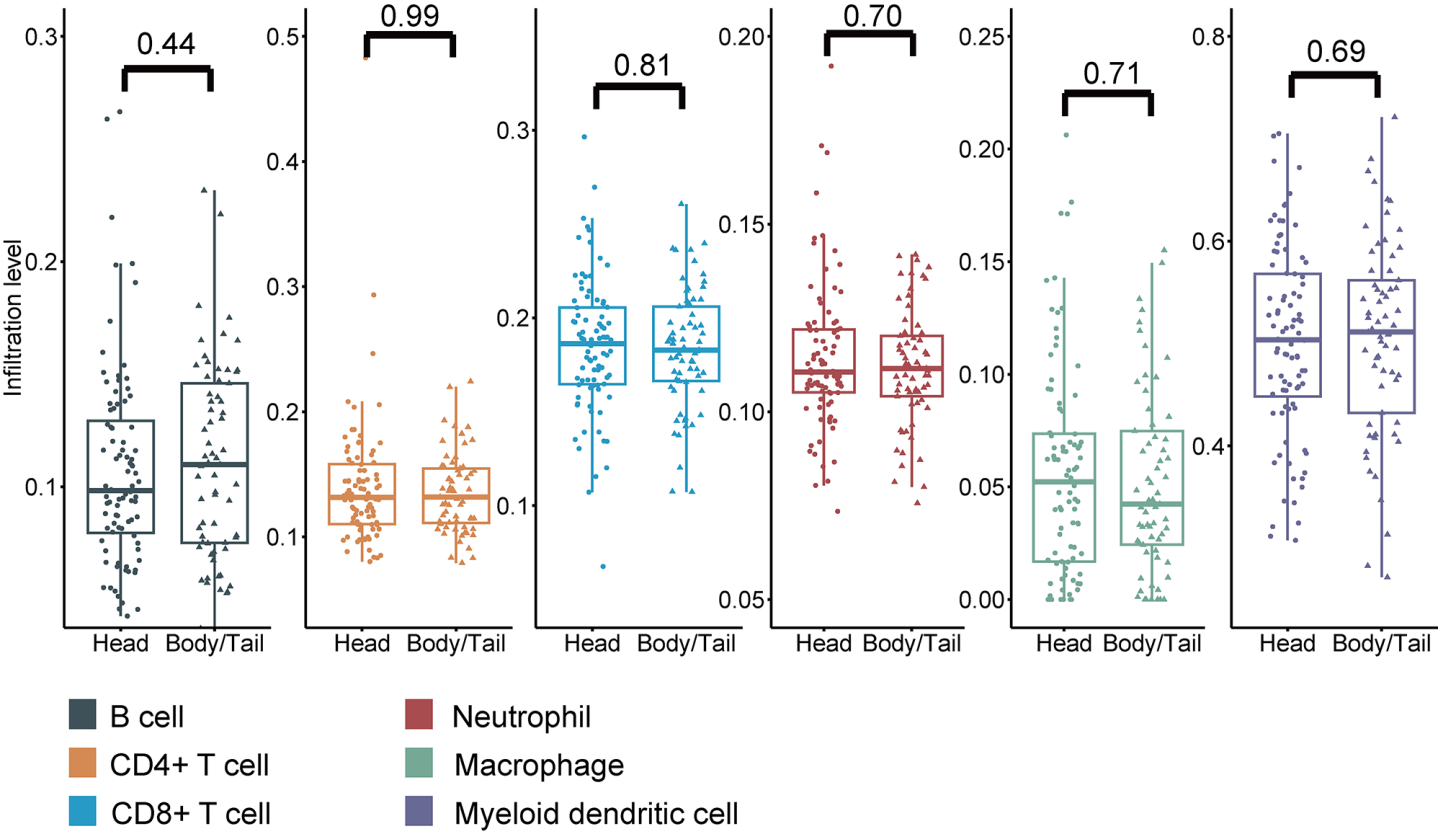
Comparison of estimated infiltration of various immune cells between pancreatic head and body/tail cancer

Using Tumor Immune Estimation Resource 2.0, the infiltration levels of B cells, CD4 + T cells, CD8 + T cells, neutrophils, macrophages, and myeloid dendritic cells were retrieved from patients with pancreatic head and body/tail cancers. There were no significant differences in the infiltration levels of the six immune cell types between the two groups (Fig. 4).

## Discussion

Pancreatic head and body/tail cancers show differences in both embryonic genesis and clinical characteristics. It is known that pancreatic body/tail cancer is associated with a poor prognosis; nevertheless, several investigations have shown inconsistent results regarding the prognostic power of tumor location [24]. This study presents the outcomes of patients with PDAC who underwent upfront surgery according to tumor location and genomic characteristics with clinical details often overlooked in many other studies. We found that tumor location was not an independent prognostic factor in patients with PDAC, and that there were no genomic differences between pancreatic head and body/tail cancers with similar clinical characteristics, including demographics and pathological stages.

Previous clinical studies have reported inconsistent outcomes regarding the effect of tumor location on the survival of patients with PDAC. Lee et al. (2020) reported that tumor location was not an independent prognostic factor, and the better survival outcomes of patients with pancreatic head cancer compared to those with body/tail cancer were associated with higher resection rates [25]. In contrast, a meta-analysis by Tomasello et al. (2019) reported that tumor location in the head of pancreas at the time of diagnosis is a significant predictor of better outcomes compared to that in body/tail (HR, 0.96; 95% CI, 0.92–0.99; *P* = 0.02) [24]. However, this study targeted a total of 93 studies, and most of the individual studies reported that tumor location was not a significant prognostic factor, and there was also a large degree of variability among the studies (*I*^2^ = 68%). In this study, which included 800 patients who underwent surgery, tumor location in the body/tail of the pancreas was identified as a significant predictor of better survival compared to that in the head in the univariate analysis but not in the multivariate analysis. We think that more frequent lymphatic and perineural invasion in pancreatic head cancer compared to pancreatic body/tail cancer may have added to the prognostic significance of tumor location in the univariate analysis. But the most important thing that caused the prognostic relevance of tumor location in the univariate analysis may be the difference in the completion rates of adjuvant chemotherapy, which was the strongest predictive factor in the multivariate analysis. The differences in surgical load between the two groups, which are supported by differences in operation type, operation time, estimated blood loss, and post-operative complication rates, may be responsible for the disparity in the completion rates of adjuvant chemotherapy.

Several studies have reported that PDAC exhibits considerable heterogeneity with a wide range of genomic alterations and gene expression [8, 9, 26]. Subsequently, some studies have reported differences in tumor biology according to tumor location in PDAC; however, these results were not consistent. Sun et al. (2022) and Zhang et al. (2021) reported that pancreatic body/tail cancer has significantly more mutations involved in main driver gene alterations, such as *KRAS*, *TP53*, and *SMAD4* [10, 11]. Mutant *KRAS* drives PDAC development and promotes tumor cell proliferation via altered metabolic pathways or activation of Wnt and MAPK pathways [27]. There are also several studies about the prognostic effect of mutant *KRAS* status [7]. Maddalena et al. (2021) reported that *TP53* missense mutations may contribute to worse PDAC prognosis by promoting a more aggressive tumor microenvironment and reducing CD8 + T cell infiltration [28]. As such, alterations in main driver genes play important roles not only in tumor initiation but also in clonal expansion processes and are also associated with worse prognosis. However, in studies conducted by Sun et al. (2022) and Zhang et al. (2021), there were significantly more patients with later stage (stage III or IV) pancreatic body/tail cancer than those with head cancer [10, 11]. Because of these differences in clinical stage, it may be challenging to interpret whether genomic differences are actually caused by the location of the tumor or its clinical severity.

In addition to somatic mutations, Dreyer et al. (2018) reported the association of tumor location in the pancreatic body/tail with squamous subtype defined by Bailey et al. (2016) [9, 29]. Abdelrahim et al. (2022) reported that patients with pancreatic body/tail cancer showed significantly lower infiltration of immune cells, including B cells, CD8 + T cells, NK cells, and neutrophils [30]. However, in Abdelrahim et al. (2022), there were significantly more distant metastasis in patients with pancreatic head cancer than in those with pancreatic body/tail cancer [30]. These findings imply that pancreatic head cancer is comparatively more susceptible to immune checkpoint inhibitors and that pancreatic body/tail cancer is less responsive to cytotoxic chemotherapy, indicating the importance of timely surgery for prognosis. Although many clinical trials on the efficacy of neoadjuvant chemotherapy and chemotherapy regimens, including immunotherapy, have been carried out in patients with PDAC, no noteworthy subgroup analysis results based on tumor location have been provided. This study, with its strength of similar clinical characteristics between pancreatic head and body/tail cancer and minimal confounding factors, such as batch effects, demonstrated that there were no significant differences in the mutational landscape, transcriptomic profiling, and infiltration of immune cells between the two groups. These findings eventually show that rather than tumor biology, disparities in prognosis according to tumor location can be induced by clinical variations, such as the timing of diagnosis and surgical loads.

We acknowledge that our study had a few limitations. First, this was a retrospective cohort study, which may have introduced a potential selection bias. Second, although deconvolution analysis with transcriptomic data from bulk tissue was used to estimate the infiltration of immune cells, transcriptome information containing a single cell unit and spatial information should be produced for more precise analysis. Third, although it was pertinent to the objective of this study, we only included patients who underwent curative pancreatectomy. Although, there were no significant differences in the proportion of patients who underwent palliative resection between patients with pancreatic head cancer (5.8%, 31/531) and those with pancreatic body/tail cancer (7.7%, 25/325), it would be preferable to include patients who did not undergo surgery in follow-up studies to lessen selection bias.

## Conclusion

In summary, we could not find differences in tumor biology and prognosis depending on tumor location in PDAC. Therefore, it is challenging to develop a treatment plan based solely on the location of the tumor, and various examinations and efforts are still needed for precision medicine in patients with PDAC.

### Electronic supplementary material

Below is the link to the electronic supplementary material.

Supplementary Material 1

## Data Availability

The whole exome and RNA sequencing data generated in this study are already available in dbGaP (https://www.ncbi.nlm.nih.gov/gap/; accession ID: phs002347.v1.p1). The clinical datasets used during the current study are available from the corresponding author on reasonable request.

## Abbreviations

PDAC: Pancreatic ductal adenocarcinoma
OS: Overall survival
HR: Hazard ratio
CI: Confidence interval
