# Supplementary material for: Comparing clinical and genomic features based on the tumor location in patients with resected pancreatic cancer
Source: BMC Cancer. 2024 Aug 26;24:1048. doi: 10.1186/s12885-024-12795-5 (PMC11346014; doi:10.1186/s12885-024-12795-5)
Supplement: Supplementary file 1 — Supplementary Material 1 [file 12885_2024_12795_MOESM1_ESM.pdf]

Supplementary Table 1 Clinical characteristics according to the treatment period

Variables	Phase I	Phase II	P
Number	284	516	
Age (years), median (IQR)	64.5 (58.0 – 71.0)	67.0 (59.0 – 74.0)	<0.001
Sex (Male)	174 (61.3)	294 (57.0)	0.270
ASA classification			0.724
I / II	261 (91.9)	469 (90.9)	
III / IV	23 (8.1)	47 (9.1)	
Symptom (Y)	197 (69.4)	331 (64.1)	0.158
CA 19-9 > 150 U/mL	146 (51.4)	233 (45.2)	0.105
Location			0.033
Head	192 (67.6)	308 (59.7)	
Body/tail	92 (32.4)	208 (40.3)	
Operation			0.039
PD	192 (67.6)	308 (59.7)	
DP/STP	91 (32.0)	207 (40.1)	
CP	1 (0.4)	1 (0.2)	
Operation type			<0.001
Open	277 (97.5)	427 (82.8)	
Minimally invasive	7 (2.5)	89 (17.2)	
Operation time (minute)	290 (195 – 371)	250 (165 – 340)	<0.001
Estimated blood loss (mL)	300 (200 – 500)	300 (200 – 500)	0.929
Complication (Y)	35 (12.3)	61 (11.8)	0.924
Examined lymph nodes > 15	125 (44.0%)	294 (57.0%)	<0.001
Stage			0.126
I	100 (35.2)	171 (33.1)	

II	124 (43.7)	260 (50.4)	
III	60 (21.1)	85 (16.5)	
R status (R0)	215 (75.7)	298 (57.8)	<0.001
Lymphatic invasion (Y)	126 (44.4)	258 (50.0)	0.146
Venous invasion (Y)	73 (25.7)	276 (53.5)	<0.001
Perineural invasion (Y)	238 (83.8)	447 (86.6)	0.325
Initiation of adjuvant CTx. (Y)	228 (80.3)	411 (80.0)	0.904
Completion of adjuvant CTx. (Y)	148 (52.1)	288 (55.8)	0.351
Adjuvant RTx. (Y)	197 (69.4)	258 (50.0)	<0.001

IQR, interquartile range; ASA, American society of anesthesiologists; CA 19-9, carbohydrate antigen 19-9; PD, pancreaticoduodenectomy; DP, distal pancreatectomy; STP, subtotal pancreatectomy; CP, central pancreatectomy; NA, not applicable; CTx, chemotherapy; RTx, radiotherapy.
